# Radiotherapy for low grade gliomas in children with neurofibromatosis type 1: when there is no other choice. Case Report

**DOI:** 10.3389/fonc.2026.1743353

**Published:** 2026-03-25

**Authors:** Serafin Castellano-Damaso, Felisa Vazquez-Gomez, Marta Perez-Somarriba, Mercedes Muñoz-Fernandez, Marta Montero-Feijoo, Ines Solís-Muñiz, Alvaro Lassaletta

**Affiliations:** 1 Pediatric Neuro-Oncology Unit, Oncology Department. Hospital Infantil Universitario Niño Jesús, Madrid, Spain; 2 Radiation Oncology Department, Hospital Universitario Gregorio Marañón, Madrid, Spain; 3 Radiation Oncology Department, Proton Center. Hospital Universitario Quironsalud, Pozuelo de Alarcón, Spain; 4 Radiation Oncology Department, Hospital Universitario de Fuenlabrada, Fuenlabrada, Spain; 5 Radiology Department, Hospital Infantil Universitario Niño Jesús, Madrid, Spain

**Keywords:** case report, low-grade gliomas, neurofibromatosis 1, pediatric, radiotherapy

## Abstract

Radiotherapy for low-grade gliomas (LGG) in neurofibromatosis type 1 (NF1) is normally avoided, since these children are at an elevated risk of developing severe sequelae. NF1 patients are more predisposed to developing radiation-induced malignancies, as well as vasculopathy. However, radiotherapy may help achieve tumor control in patients with life-threatening complications or a long history of relapse. Here we present two NF1 patients in whom radiotherapy managed to achieve full control of the disease without side effects, sparing those patients from needing further therapies. Literature regarding this aspect is also reviewed.

## Introduction

Neurofibromatosis type 1 (NF1) represents the most common tumor predisposition syndrome (1, 2). Up to 15-30% of the NF1 patients will develop brain gliomas (2–4), most commonly in the optic pathway and the brainstem (3) (5). Usually, most patients won’t need treatment (6), as they may also develop spontaneous regression of the tumors (7, 8). Chemotherapy is the preferred choice in case they finally need treatment, especially in unresectable tumors (6, 9, 10).

Radiotherapy shows excellent rates of tumor control in treating pediatric LGG (11–20), with rates of event-free survival (EFS) of around 70-80% at 10 years and 10-year overall survival (OS) of up to 90% (11, 12, 15, 20). However, it is generally avoided in patients with NF1 (21, 22), as they are predisposed to developing radiation-induced tumors (23, 24). Radiotherapy can also cause cerebral vasculopathy lesions in NF1 patients (25, 26). Nevertheless, the high rates of tumor control achieved with radiotherapy can make it an optimal salvage treatment in very selected patients with exceptional circumstances.

We present two NF1 patients with LGG treated at our institution, who in severe situations, were treated with radiotherapy for their progressive LGGs. In the first case, a glioma centered on the medulla oblongata was growing while on chemotherapy and affecting the brainstem. He received radiotherapy as an urgent life-threatening treatment. In the second case, radiotherapy was applied as a fifth-line treatment due to recurrent tumor progression after several surgeries and chemotherapy lines. In both cases, the patients achieved full control of the disease and were stable at the last follow-up.

## Case reports

### Case 1

**Figure 1:**
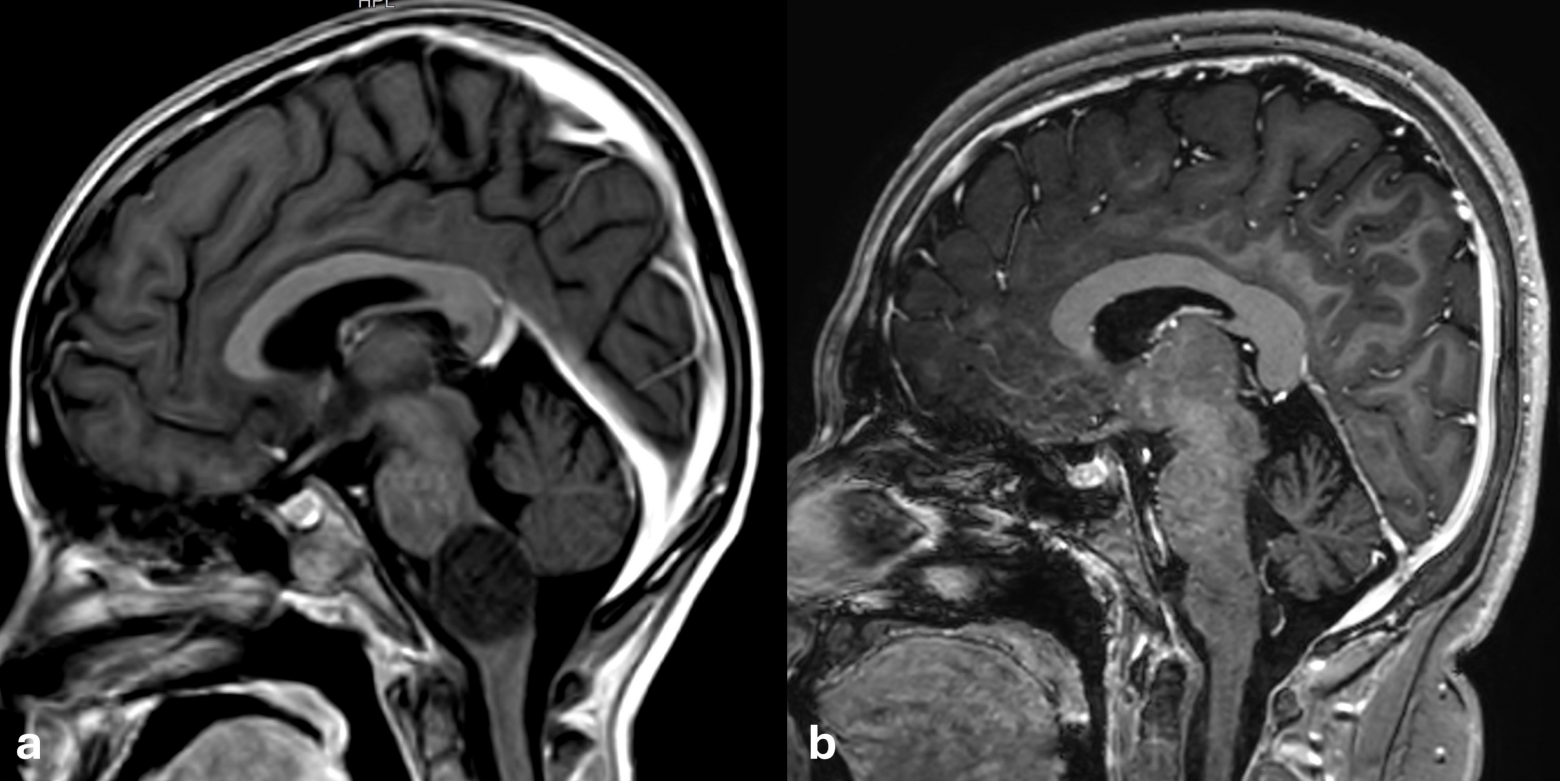
Sagittal T1-weighted MR images after contrast administration at the ages of 8 years **(a)** and the current age of 15 years **(b)** in Patient 1. The patient presented with a focal expansive tumor in the medulla oblongata, which significantly decreased in volume and recovered normal signal on T1 and T2 sequences (not shown). The lesion did not enhance after intravenous gadolinium administration.

**Figure 2:**
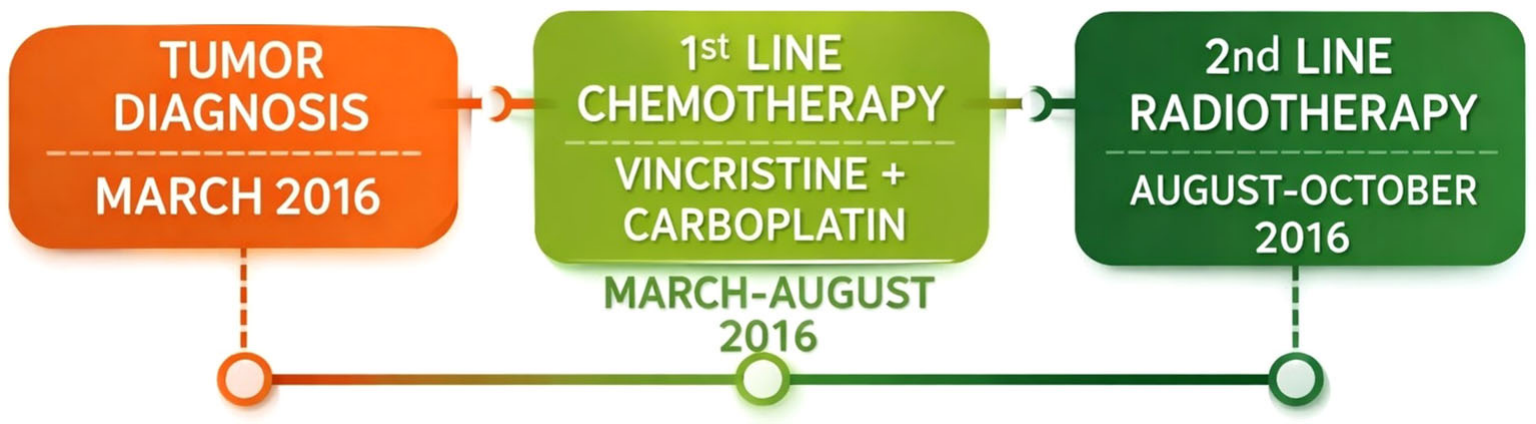
Timeline 1. Timeline of Patient 1. (Figure created with the assistance of an AI-based design tool).

A 7-year-old male, with a previous diagnosis of familial neurofibromatosis 1, was diagnosed at our institution in March 2016 with an expansive lesion centered on the medulla oblongata and a dubious synchronous millimetric lesion in the left cerebral peduncle (**Figure 1a**). At presentation, he was suffering from ataxia, dysarthria and motor slowness, and a brain magnetic resonance imaging (MRI) was requested. After the radiological diagnosis, he started first-line chemotherapy with vincristine and carboplatin. He developed grade 3 pancytopenia, as well as grade 3 hyponatremia. After five months of treatment, he developed a severe hypertensive emergency due to acute clinical tumor progression with brainstem compression, with secondary respiratory failure with hypoxemia, and the patient was admitted to PICU. While his blood pressure was successfully lowered, hypoxemia worsened in the context of tumor growth. He was intubated and started on mechanical ventilation and dexamethasone. In this emergency context with signs of brainstem compression, urgent radiotherapy was decided. He started in August 2016 3D conformal radiation-therapy and received 30 sessions of 1.8 Gy for a total dose of 54 Gy. Clinical improvement allowed the patient to be discharged after 17 sessions, with the first two sessions being given while in PICU. He still suffered from frequent hospital admissions due to hypertensive crisis and SIADH-related hyponatremia. Nevertheless, while continuing radiotherapy, his serum sodium and blood pressure started to improve as well as his clinical condition. The end-of-radiotherapy MRI showed stable disease, with partial response over the years (**Figure 1b**). After 9 years of follow-up, he has no new signs of tumor progression nor secondary long-term effects of radiotherapy, vasculopathy, secondary malignancies or evidence of radiation necrosis (**Figure 2**, Timeline 1).

### Case 2

**Figure 3:**
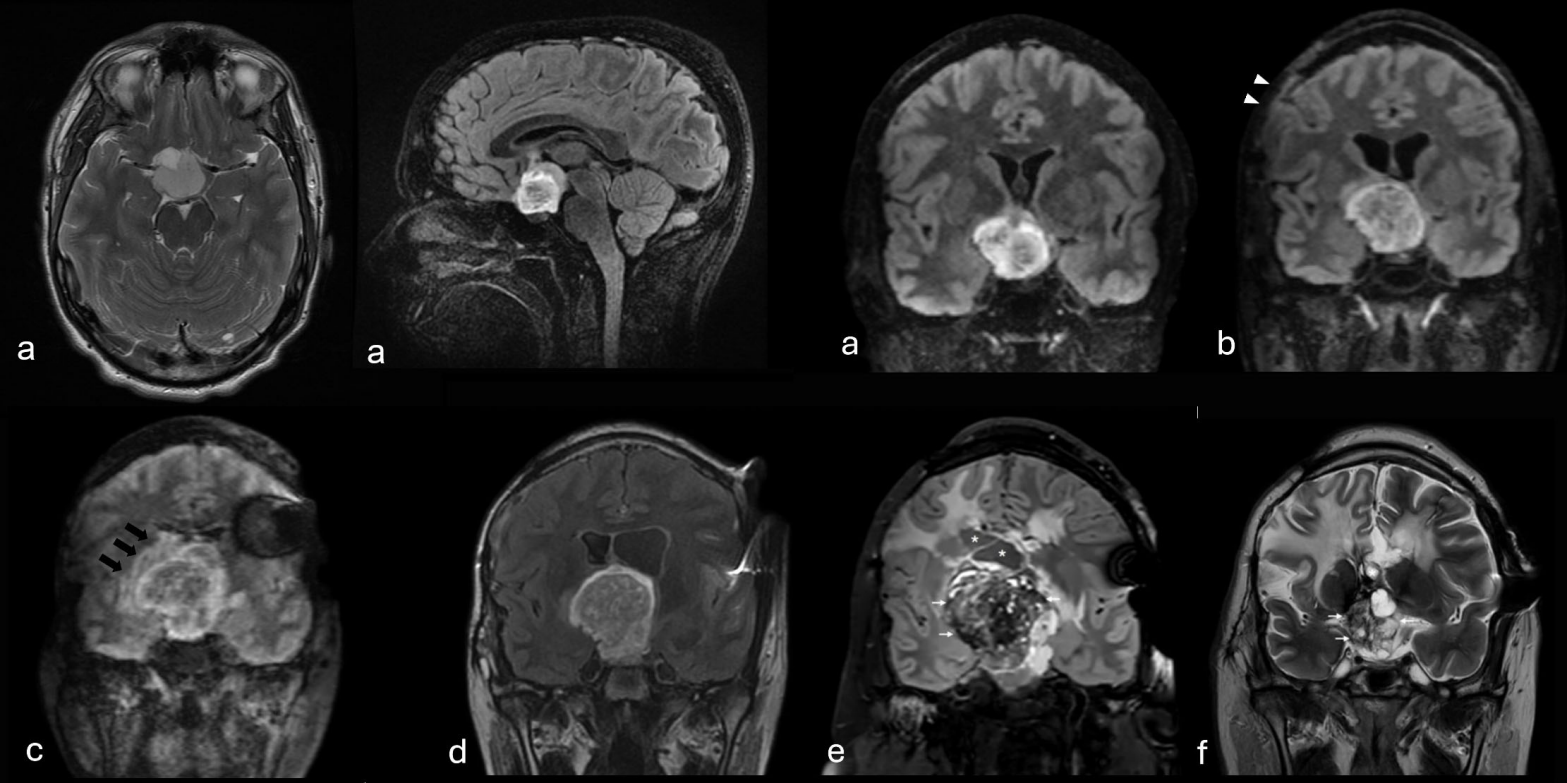
T2−weighted FLAIR MR images depicting the 7−year longitudinal evolution of Patient 2 with a solid–cystic optic pathway glioma. **(a)** At presentation, the lesion shown is predominantly solid, centered in the suprasellar cistern, involving the optic chiasm and mildly indenting the right hippocampal uncus (axial, sagittal and coronal images, respectively). **(b)** After two partial resections (2019), postoperative changes are evident (arrowheads), with increased mass effect and ventriculomegaly. **(c)** Following completion of vinblastine therapy, the tumor shows marked interval growth with increased perilesional edema (black arrows). Susceptibility artefacts from the left parietal VP shunt valve are visible. **(d)** Tumor status at the end of trametinib therapy (December, 2020). **(e)** On subsequent follow−up immediately prior to initiation of proton therapy (March, 2023), the tumor is markedly enlarged, with numerous cystic components and pronounced perilesional edema. **(f)** Coronal T2 TSE images one year later. The solid portion (arrows) showed an important decrease in size and mass effect, and the cystic portions (asterisks) at its most cranial margin were almost completely reduced.

**Figure 4:**
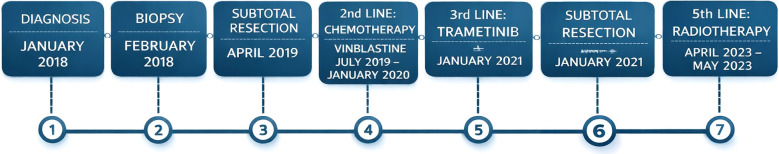
Timeline 2. Timeline of Patient 2. (Figure created with the assistance of an AI-based design tool).

A 17-year-old male with a diagnosis of familial NF1 from the age of three was diagnosed in January 2018 with a large suprasellar midline tumor involving the anterior optic pathway. He underwent a biopsy at another institution and started observation in February 2018 (**Figure 3a**). Following tumor progression one year later, a subtotal resection was pursued at the referral center. Partial resection was achieved in April 2019, which led him to right eye blindness (**Figure 3b**). While surgery is not generally considered to be a first-line option for this kind of large midline gliomas due to the high risk of sequelae secondary to the surgery, in this case it was performed due to hydrocephalus concerns at the referral center. As consolidation chemotherapy, he received weekly vinblastine for 6 months, stopping it when he required a ventriculoperitoneal (VP) shunt due to new tumor progression and hydrocephalus (**Figure 3c**). As third-line treatment he started empirically MEK-inhibitor trametinib in May 2020 and was stopped after 7 months due to tumor progression (**Figure 3d**) which required a new subtotal resection in January 2021. After this surgery, he suffered severe panhypopituitarism with full hormonal replacement. He also suffered from a VP-shunt infection, a pulmonary embolism, and a second VP-shunt replacement due to valve obstruction. His central line also needed to be replaced due to repeated episodes of *Klebsiella oxytoca* bacteremia. He also complained of daily headaches, memory impairment as well as minor left-sided hemiparesis, and he was referred to our institution at new tumor progression (**Figure 3e**). Because of all these surgical and medical complications, and with tumoral progression to several lines of treatment, it was decided to start focal radiotherapy. At the referral center, radiotherapy was initially not given due to his condition of neurofibromatosis type I. In this NF1 setting, we decided to offer the patient proton therapy because the lesion was large and the radiation volume would also be substantial, in order to minimize exposure of healthy tissue to high radiation doses, specifically in this patient with previous comorbidities. Proton radiotherapy performs favorably and with fewer side effects in pediatric patients, especially in the long term. After discussion in our Brain Tumors’ Board, PBT was selected, and he started protons in April 2023, up to a total dose of 50.4 Gy in 1.8 Gy fractions. During radiotherapy, he was admitted twice to hospital due to PICC-line infection for intravenous antibiotics, with no other complications. He experienced a partial radiological response at 8 months after finishing PBT (**Figure 3f**). Clinically, the patient’s clinical condition has improved over time, with improved self-well-being. His neurocognitive status has been recovering, with evidence of short-term memory amelioration, absence of headaches and motor improvement. As with our first patient, he has been clinically improving, and maintaining the partial response at last follow-up, almost 3 years after completing radiotherapy, with no signs of pseudoprogression, second malignancies, vasculopathy nor radiation necrosis so far (**Figure 4**, Timeline 2).

## Discussion

LGGs comprise a heterogeneous entity of low-grade glial tumors with an excellent prognosis, with OS rates of almost 90% at 20 years (27). Regarding NF1, up to 30% of NF1 patients may develop LGG (4), especially in the optic pathway and the brainstem (3), with pilocytic astrocytomas being the most usual histology in NF1 patients (1, 2). Neurofibromatosis type 1 patients with low-grade gliomas also experience excellent OS, with higher EFS than non-NF1 patients (28), being especially true in optic pathway gliomas (OPG), with better outcomes in the NF1 patients (29). Common treatments for treating low-grade gliomas in these patients include surgery if a safe resection is possible, and chemotherapy in unresectable tumors (6, 9), with radiotherapy not being a usual first-line treatment. Regarding diagnosis, a biopsy is not always required in patients with NF1 when MRI findings are consistent with an optic pathway glioma. In our second case, an initial biopsy, and then a partial resection resulted in permanent loss of vision in the right eye—an outcome that could likely have been avoided, as the procedure did not alter subsequent management or overall outcome.

Nevertheless, radiotherapy has been widely used as part of the LGGs’ treatment in the non-NF1 patients, both as first-line and in the relapse/progression setting (11, 13, 14). It is a safe and effective technique, with excellent rates of local tumor control. This was the case of our second patient, who got a partial response after 8 months of finishing radiotherapy and improved his clinical condition. Radiation therapy managed to get the best results so far after all his previous treatment. However, despite access to modern radiotherapy techniques such as protons (20), most pediatric neuro-oncologists avoid radiotherapy in the initial treatment of pediatric LGGs due to increased risk of stroke, secondary brain tumors, endocrine deficits, and decline in cognition in younger patients (20, 30–34). This is even more important in the NF1 population, where the use of radiotherapy is questioned because of these side effects (21–26). NF1 patients are more likely to develop second cancers in the radiation field due to the constitutional loss of the tumor suppressor *NF1* gene (35). This makes them more prone to the appearance of radiation-induced tumors such as high-grade gliomas (HGG) and malignant peripheral nerve sheath tumors (MPNST), being more aggressive and resistant to conventional treatment (23, 24). These patients are up to a threefold increased risk increased risk of developing radiation-induced HGG (24), with poor prognosis. In the work by Bhatia et al. (33), they analyzed two large cohorts of patients. The first one, the Childhood Cancer Survivor Study, yielded a 2.4-fold higher risk of secondary malignancies in the NF1 patients compared to non-NF1 patients at 20 years of follow-up. The second one, the University of Alabama at Birmingham and Children’s Hospital of Philadelphia cohort, showed that the risk of secondary tumors after 10 years of follow-up was 2.8-fold higher in NF1 patients who received radiotherapy compared to those who did not. After excluding plexiform neurofibromas, this risk was even higher, with a 6.1-fold higher risk of developing secondary cancers in the radiated group. Moreover, in the review by Madden et al. (24), four patients developed HGG after radiation therapy for a primary low-grade glioma, with none of them surviving more than 24 months after diagnosis. Prognosis in radio-induced MPNST is also dismal. MPNSTs are malignant tumors common to NF1 patients, with a fortyfold increased risk of developing these tumors after receiving radiotherapy (34). MPNST represents the most common type of radiation-induced malignancy (24), with radiation-induced MPNST being of worse prognosis than sporadic and NF1-related MPNST (34, 36), with median OS of 24 months (34).

Furthermore, the loss of neurofibromin, the protein product of the *NF1* gene, in the endothelium causes these patients to develop vasculopathy over time (37–39). It could worsen if radiotherapy is applied, being highly deleterious if radiation is given to the brain vessels, causing bleeding, thrombosis, or moyamoya phenomenon (38). Grill et al. described a series of sixty-nine patients irradiated for OPG, both NF1 and non-NF1 (25). Thirteen patients developed radiation-induced vasculopathy, with 11 of those 13 patients (84.6%) being neurofibromatosis type 1 patients, with an almost 5-fold risk of developing vasculopathy. Similar findings were those published by Ullrich et al. (32): a total of 345 children underwent radiotherapy for treating several primary brain tumors, of which nineteen of them had a previous diagnosis of neurofibromatosis type 1. Twelve of those patients developed moyamoya phenomenon, of which four were prior NF1 patients. As in the previous work, NF1 patients may be up to five times more likely to suffer from cerebral vasculopathy when exposed to radiation. The impact of NF1 in developing moyamoya was also described by Wang et al (31). Their work implied a shorter time interval between radiotherapy and diagnosis of moyamoya between NF1 patients and non-NF1 patients (3.2 vs. 4.2 years). Bavle et al. (30), reported NF1 as a significant risk factor for vasculopathy and stroke after photon radiation for pediatric brain tumors, with rates of up to 60% of vasculopathy in NF1-patients. However, Tsang et al, in the description of 89 OPG patients with fourteen patients being NF1, did not find an increased risk of developing vasculopathy after radiotherapy. It did show however a higher risk for second malignancies outside the radiation field (40). Other side effects of radiotherapy in the NF1 population include neurocognitive impairment as well as endocrine disorders (25).

Focusing on PBT, it has been generally considered to be a technique with a more amenable side effects profile (41). Although there have been some recent concerns regarding a greater risk of vasculopathy in patients who have received PBT, this finding seems to be controversial. In the systematic review by Elkatatny et al. it is described that PBT causes a greater risk of moyamoya (42), with an incidence of 9.3% of moyamoya among patients who received PBT (although only one in the series had a diagnosis of NF1, being just a 3.5% of the total number of patients affected with moyamoya) compared to an incidence of 5% among patients who received photon radiotherapy, with a total of 7 patients being NF1 and representing 16.7% of the total number of patients. Thus, according to the data of this publication, radiation-induced moyamoya in NF1 patients was more common among children who were treated with photons. While it is true that in this paper there could be a selection bias due to the tendency to publish more patients who have suffered from vasculopathy after receiving a relatively new technique of radiation such as PBT (and with a larger population of patients receiving radiotherapy with photons), other works describe a similar risk of moyamoya of around 3-5% in patients who have been treated with protons. This incidence is similar to the one developed after conventional photon radiation therapy, with a lower rate of 2.4% in the work by Gill et al. (43). In this review, the authors found 14 patients who developed vasculopathy among a retrospective cohort of 676 pediatric patients who received protons for treating a variety of brain tumors. The authors described as risk factors of developing radiation-induced moyamoya syndrome a higher dose given to the circle of Willis and optic chiasm. This was especially true in patients who have received a dose >52 Gy to the optic chiasm, while they were unable to determine the risk dose to the circle of Willis. In the described population, age was not considered to be a risk factor, as the age cutoff point was not related to an increased risk of radiation-induced vasculopathy. All in all, PBT may produce a higher risk of moyamoya in small children, specifically during the first years after radiotherapy. This was not the case of our 2^nd^ patient, as he received proton beam therapy as a young adult, and nearly 3 years after finishing radiotherapy he has not developed any signs of vasculopathy so far.

Despite the described risks, there are critical and urgent cases, where radiotherapy can be of help in treating LGG in NF1 patients. LGGs tend to grow slowly over time (44), but sometimes this slow growth could be harmful to vital structures specially on the brainstem (45). Radiotherapy can help achieve faster tumor control and may induce a longer remission period (11). In the review by Ajithkumar et al., OS and EFS for LGG treated with radiotherapy remains high as depicted previously, being also true for NF1 patients. For instance, the 10 NF1 patients of the German cohort who received radiotherapy achieved a 5-year EFS of 78%, which was better than the chemotherapy cohort (46). None of those patients suffered from any late effects, although the follow-up period may not have been long enough. These findings were similar to the work by Wentworth et al., with a 5-year progression-free survival (PFS) of 75% (22), as well as those by Tsang et al., with 10-year EFS of 61.9% and 10-year OS of 92.3% (40). PBT, despite being a relatively new technique, has also been used in the treatment of pediatric LGG, with also high PFS and OS rates, as reported by Indelicato et al., with 5-year actuarial rates of 84% and 92% (20). This work included three patients with NF1, with no detailed reporting regarding their outcome. Regarding quality of life, PBT also performs favorable. In the review by Willmann et al. published in 2023 (41), 89 patients (including 4 cases with a previous diagnosis of NF1) were retrospectively analyzed, and quality of life was prospectively assessed. Patients were compared in different aspects of quality of life (including sexual health) to the reference value, during PBT and for six years after. It was found that these patients were comparable and similar to the reference population.

Our two patients received radiotherapy at distinct stages, in the first patient given urgently due to accelerated tumor growth and in the second patient as fifth-line treatment due to recurrent tumor progression. LGGs recur frequently, especially in those tumors that cannot be resected at diagnosis (44, 45). Surgery in these situations could be potentially life-threatening, or may leave the patient with unacceptable morbidity, such as motor dysfunction which causes life-long disabilities, loss of visual acuity, or hormone deficiencies (27, 47, 48), as was the case of our second patient.

In conclusion, radiotherapy (preferably PBT if given the opportunity and after careful discussion in a multidisciplinary team meeting) may represent an adequate option for treating patients with NF1-associated low-grade gliomas in very selected situations. However, the decision about whether to radiate a patient should be carefully considered. Risk-benefit balance must be carefully pondered, and the potential long-term damage also needs to be reflected in the final decision.

## Data Availability

The raw data supporting the conclusions of this article will be made available by the authors, without undue reservation.
